# 11. AEROBIC EXERCISE TRAINING FOR INDIVIDUALS WITH SCHIZOPHRENIA: THE BROAD BENEFITS ACROSS PHYSICAL HEALTH, COGNITION, AND EVERYDAY FUNCTIONING AND PROMISING MECHANISMS OF ACTION

**DOI:** 10.1093/schbul/sby014.038

**Published:** 2018-04-01

**Authors:** Keith Nuechterlein

**Affiliations:** University of California, Los Angeles

## Abstract

**Overall Abstract:** Recently aerobic exercise training has begun to be systematically examined in randomized controlled trials (RCTs) in schizophrenia. This symposium will report and discuss the results of RCTs that examined the impact of aerobic exercise on physical health, cognition, and everyday functioning across first-episode and established illness phases of schizophrenia. In addition, data on neurotrophic and brain structural changes will be examined as promising mechanisms of action. Dr. Amal Abdel-Baki of the University of Montreal has focused on the physical health benefits of interval training in her RCT with first episode and multi-episode schizophrenia outpatients. She is demonstrating improved waist circumference, diastolic blood pressure, HDL cholesterol, and social functioning in first episode and multi-episode patients. Dr. David Kimhy of Icahn School of Medicine at Mount Sinai in New York has focused on the impact of aerobic exercise training on cardiovascular fitness, Brain-Derived Neurotrophic Factor (BDNF), cognition, and functional outcome in individuals with an established schizophrenic illness. He has demonstrated beneficial effects at each of these levels. Furthermore, relationships between fitness improvements and BDNF increases and the cognitive and functional gains suggest potential mechanisms of action. Dr. Berend Malchow of Ludwig Maximilian University of Munich has examined the impact of adding cognitive training to aerobic exercise in multi-episode schizophrenia patients. This combination led to increased verbal memory and improved global functioning. Increase in left temporal grey matter volume is a promising brain mechanism of action. Dr. Keith Nuechterlein from UCLA will present results from a recently completed RCT of first-episode schizophrenia patients in which aerobic exercise training was added to computerized cognitive training to determine the extent to which it could enhance the impact of cognitive training. He will show that this combination significantly enhances cognition and work/school functioning gains beyond the effect of cognitive training alone and leads to increases in prefrontal cortical thickness and functional connectivity. Furthermore, he will examine early BDNF increases in response to treatment as a predictor of later cognitive and functional improvements. Dr. Peter Falkai will lead the discussion of the promise of aerobic exercise as an intervention to improve physical health, cognition, and functional outcome in schizophrenia and consider the potential mechanisms of action.

